# Protein Tyrosine Phosphatases: Mechanisms in Cancer

**DOI:** 10.3390/ijms222312865

**Published:** 2021-11-28

**Authors:** Vignesh Sivaganesh, Varsha Sivaganesh, Christina Scanlon, Alexander Iskander, Salma Maher, Thư Lê, Bela Peethambaran

**Affiliations:** 1 Department of Biological Sciences, University of the Sciences, 600 S 43rd St, Philadelphia, PA 19104, USA; vsivaganesh@mail.usciences.edu (V.S.); varshasivaganesh@gmail.com (V.S.); cscanlon@mail.usciences.edu (C.S.); aiskander@mail.usciences.edu (A.I.); smaher@mail.usciences.edu (S.M.); tle3000@mail.usciences.edu (T.L.); 2 Department of Biomedical Sciences, Philadelphia College of Osteopathic Medicine, 4170 City Ave, Philadelphia, PA 19131, USA

**Keywords:** breast cancer, gastric cancer, prostate cancer, PTP, protein tyrosine phosphatase, protein tyrosine kinase, receptor tyrosine kinase, oncogene, tumor suppressor

## Abstract

Protein tyrosine kinases, especially receptor tyrosine kinases, have dominated the cancer therapeutics sphere as proteins that can be inhibited to selectively target cancer. However, protein tyrosine phosphatases (PTPs) are also an emerging target. Though historically known as negative regulators of the oncogenic tyrosine kinases, PTPs are now known to be both tumor-suppressive and oncogenic. This review will highlight key protein tyrosine phosphatases that have been thoroughly investigated in various cancers. Furthermore, the different mechanisms underlying pro-cancerous and anti-cancerous PTPs will also be explored.

## 1. Introduction

Protein tyrosine phosphatases (PTPs) function to remove phosphate groups from proteins. Historically, PTPs have been thought of as being opposite to protein tyrosine kinases (PTKs), since PTKs are enzymes that add phosphate groups to proteins. One major focus of cancer research involves understanding how a subset of PTKs, known as receptor tyrosine kinases (RTKs), enables the progression and survival of cancer. RTKs, many of which are growth factor receptors, have intracellular domains that auto-phosphorylate upon ligand binding [1,2]. This induces the activation of kinases that phosphorylate downstream proteins, which can further phosphorylate even more downstream proteins [1,2]. The phosphorylation cascade results in the activation of many normal cell signaling pathways that induce proliferation, differentiation, survival, and cell migration [1,2]. Some important growth-promoting signaling proteins, which are recruited to the activated RTK’s intracellular phosphotyrosine residues, include phosphoinositide 3 kinase (PI3K), Ras, and Janus kinase (JAK) [1,2,3]. PI3K, Ras, and JAK generate the PI3K-AKT, mitogen-activated protein kinase/extracellular signal-regulated kinase (MAPK/ERK), and JAK-STAT signaling cascades, respectively [1,2,3]. Therefore, upregulation of RTK expression is a key molecular mechanism that underlies many cancers. On the other hand, PTPs were thought to inhibit the actions of RTKs by performing the reciprocal action of kinases, which is to dephosphorylate proteins [4,5]. However, it is now known that in normal physiology, some PTP dephosphorylation balances RTK and PTK phosphorylation to regulate cell signaling dynamics, while other PTP subtypes are known to be proto-oncogenic by enhancing growth factor and survival signaling pathways [4,5].

Protein tyrosine phosphatases can be classified as tyrosine-specific or dual-specific [4,5,6]. Dual-specific PTPs are able to dephosphorylate both tyrosine and serine/threonine residues [4,5,6]. Furthermore, PTPs can also be grouped based on their location within the cell. PTPs that span the cell membrane are receptor types, while PTPs within the cytosol are non-receptor types [4,5,6]. Despite these classifications, PTPs exhibit oncogenic or tumor-suppressive activity via their ability to dephosphorylate proteins [4,5]. It is the combination of PTP and the unique pathway it operates on that determines whether dephosphorylation will lead to promotion or hindrance of tumorigenesis. Hence, a given PTP can have the same function or different function across many organs. A PTP that exhibits loss of function in one cancer can have a gain of function mutation in a different cancer.

Some heavily studied PTPs can provide insight into the general mechanisms of tumor-suppressive and oncogenic PTPs. Src homology-2-containing protein tyrosine phosphatase 2 (SHP-2) is a non-receptor, tyrosine-specific, PTP encoded from the PTPN11 gene that binds to the RTK intracellular domain via phosphotyrosine residues [7,8,9]. The Src homology 2 domain of SHP-2, once occupied by tyrosine residues, no longer impedes the phosphatase activity of SHP-2 [7,9,10]. SHP-2 dephosphorylates certain proteins or residues that suppress the function of Ras inhibitors and Src family kinase (SFK) inhibitors, thus enhancing the activity of these tumorigenic pathways [11,12,13,14]. In other words, SHP-2, through its ability to dephosphorylate and inactivate SFK and Ras inhibitors, can promote the PI3K and MAPK/ERK signaling that arises downstream of activated RTKs [11,12,13,14,15,16,17,18]. SHP-2 is an example of a PTP that seems to exhibit oncogenic activity in many cancer cell types, which will be covered later in the review.

Phosphatase of regenerating liver 3 (PRL-3), also known as PTP4A3, is a dual-specific, non-receptor PTP that is highly expressed in many tumors [6]. Upon activation of growth factor receptors like platelet-derived growth factor (PDGF), Src, a tyrosine kinase that is a part of the Src family of kinases, phosphorylates and activates PRL-3 at specific amino acid residues [19]. Once activated, PRL-3 can act upstream of Src kinase by downregulating expression of Src kinase inhibitor c-Srk kinase (Csk), thereby promoting the oncogenic RAS-RAF-MEK-ERK pathway downstream of Src kinase [20]. PRL-3, like SHP-2, also exhibits tumor-promoting activity across different cancer types.

In contrast, protein tyrosine phosphatase 1b (PTP1b), a non-receptor, tyrosine-specific PTP, has been implicated in both hindering and promoting cancer [6]. In studies that were mainly done in fibroblasts and endothelial cells (cell types that can enhance tumorigenesis), inhibition of PTP1b proved to enhance migration, while overexpression of PTP1b suppressed migration [21,22]. One of the major molecular mechanisms underlying PTP1b’s ability to reduce migration is the dephosphorylation and inactivation of p130cas, which impedes the scaffold protein’s ability to recruit and activate Rac [23,24,25]. The Rac signaling pathway is crucial in promoting actin polymerization and lamellipodia formation during cell migration [26]. Rac signaling can also activate matrix metalloproteinases that can degrade the extracellular matrix to promote cancer invasion [23,27]. Thus, the tumor suppressor function of PTP1b is highlighted through its ability to dephosphorylate key proline-rich residues of p130cas and inhibit p130cas-Rac induced migration and invasion of fibroblasts and endothelial cells [25].

PTP1b can also exhibit oncogenic activity in many cancers. One study found that increased PTP1b expression was associated with poor survival in pancreatic ductal adenocarcinoma (PDAC) patients and that overexpression of PTP1b in PDAC cell lines promoted aggressive proliferation and migration [28]. These results were further corroborated by in vivo studies that identified a marked reduction in tumor growth with PTP1b knockdown or pharmacological inhibition of PTP1b [29]. In this study, the mechanism of PTP1b-induced PDAC aggression involved the modulation of the pyruvate kinase M2 (PKM2) pathway [29]. PKM2 is a protein kinase that plays an important role in glucose metabolism and phosphorylation of other proteins implicated in malignancy [30]. PKM2 is an important regulatory junction that allows PTP1b to act as an oncogene.

PTP1b continues to be identified as a tumor promoter, with recent evidence emerging in glioblastoma-multiforme, colorectal cancer, and ovarian cancer. One study identified that PTP1b, through its association with Interleukin 13 Receptor alpha 2 (IL13Rα2), induced Src activation via dephosphorylation of Tyr527/530 residues (phospho-Tyr527/530 inhibits the SH2 domain from binding to RTK phosphotyrosine residues) and promotes downstream PI3K/AKT and Ras-Raf-Mek-Erk signaling [17,18,31,32,33]. By suppressing this pathway through PTP1b inhibition, researchers identified a reduction in migration, invasion, and proliferation in glioblastoma-multiforme (GBM), colorectal cancer, and ovarian cancer cell lines [31]. These results were further corroborated by associating heightened PTP1b expression in the three types of cancers with lower overall patient survival; the study also utilized PTP1b inhibitor to markedly reduce tumor growth and increase survival rates in mice inoculated with GBM cells [31]. Though these findings identify PTP1b as a promising cancer-specific molecular target, the dual oncogenic and tumor-suppressive nature of PTP1b may cause future complications. In addition to PTP1b’s tumor suppressor activity in endothelial cells and fibroblasts, PTP1b has also been identified as a negative regulator of insulin-like growth factor receptor (IGFR) signaling that attenuates kinase signaling associated with survival and metastasis in ovarian cancer [34]. However, strong evidence of PTP1b’s function in PDAC, GBM, colorectal cancer, ovarian cancer, and breast cancer mentioned later in this review suggests that while PTP1b promotes oncogenesis in cancer cells, it may be a tumor suppressor in fibroblasts and endothelial cells.

In addition to non-receptor PTPs, including the previously mentioned PTP1b, PRL-3, and SHP-2, there exists a group of small PTPs generated from one human ACP1 gene [6,35]. The ACP1 gene is capable of producing a few PTP isoforms. These small 18 kDa PTPs, known as low molecular weight PTPs (LMW-PTP), can interact with receptor tyrosine kinases, non-receptor kinases, and other proteins that regulate metastasis, growth, and apoptosis, resulting in both pro-tumorous and anti-tumorous capabilities [35]. Another type of PTP that spans the cell membrane, known as receptor protein tyrosine phosphatase (RPTP), was once thought of as a receptor that antagonizes the cancerous signaling pathways activated by RTKs [36]. Emerging research suggests that RPTPs are implicated in promoting and hindering cancer formation. Unlike LMW-PTPs, different genes give rise to a variety of the RPTPs [36].

In comparison to protein tyrosine kinases, not much is known about PTPs. However, there have been many studies performed on the wide variety of PTPs that regulate molecular pathways related to cancer. In this review, we will highlight research that has identified potential oncogenic or tumor-suppressive roles of different PTPs via their influence on PI3K-AKT, Ras, and other crucial cancer-signaling cascades. We hope this review will enable scientists to understand the detailed mechanism of PTPs and identify new PTP actions, as well as PTP targeting therapeutics across a wide range of cancers.

## 2. PTPs That Regulate the JAK-STAT Pathway

**Figure 1:**
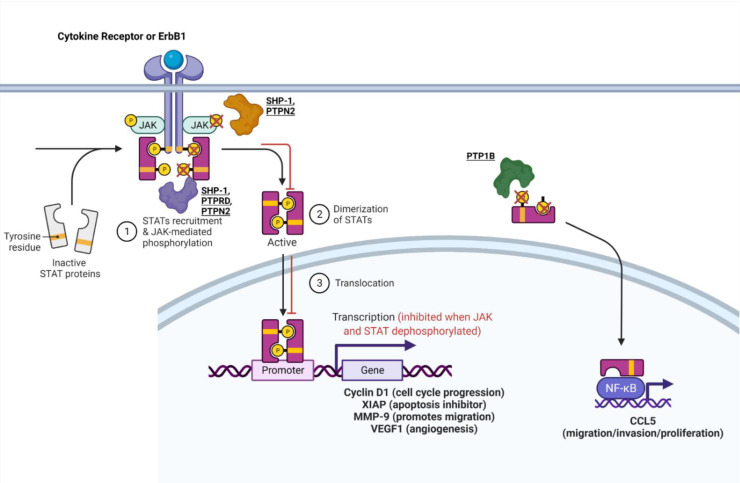
**PTPs that regulate the JAK-STAT pathway.** The mechanism of some PTPs influence the JAK-STAT or STAT-CCL5 pathway. For a more in-depth discussion of the mechanism of action for each PTP, refer to Table 1. Adapted from “Cytokine Signaling through the JAK-STAT Pathway”, by BioRender.com (accessed on 28 September 2021). Retrieved from https://app.biorender.com/biorender-templates (accessed on 28 September 2021).

Emerging research suggests that PTPs play a key role in regulating different aspects of the JAK-STAT pathway. SHP-1, a non-receptor, tyrosine-specific PTP that exhibits tumor suppressor activity in gastric cancer, was found to dephosphorylate and inhibit STAT3 signaling in vitro and in vivo [37]. Upon increasing SHP-1 expression, cell proliferation was inhibited in vitro, and tumor growth was inhibited in vivo [37]. Additional experiments led researchers to discover that when SHP-1 protein expression was increased, gastric cancer cells exhibited reduced phosphorylation of STAT3, which resulted in downstream inhibition of Cyclin D1 and XIAP (Figure 1) [37]. Furthermore, another study identified that SHP-1 gene expression is reduced in gastric cancer cells owing to promoter region hypermethylation [38]. Overexpression of SHP-1 in gastric cancer cells downregulated the JAK-STAT pathway (decreased phosphorylation of JAK2 and STAT3 due to its dephosphorylation activity) and led to the downregulation of proteins that are important for cell cycle progression, invasion, and angiogenesis (Cyclin D1, MMP-9, VEGF1) (Figure 1) [38]. Both studies identified key molecular mechanisms underlying SHP-1′s ability to inhibit cancer progression, which provides strong evidence that SHP-1 is a tumor suppressor (Table 1). Further research is needed to identify SHP-1 loss of function in gastric cancer as a predictor of poor prognosis and decreased patient survival.

Protein tyrosine phosphatase 1b (PTP1b) promotes tumorigenesis in many types of cancer including breast cancer. It was shown to be overexpressed primarily in HER2+ breast cancers and influence the JAK-STAT pathway [28]. In one study, PTP1b increased tumor size and lymph node metastasis by dephosphorylating phospho-STAT 3, thus increasing CCL5 expression, which is involved in increased cell migration and proliferation (Figure 1) [28]. This finding contradicts the known function of the STAT3 pathway, in which dephosphorylated STAT3 is inactive and typically inhibits tumorigenic pathways. However, the researchers in this study confirmed their findings through the knockdown of PTP1b, which increased phosphorylation of STAT3, resulting in decreased CCL5 expression and diminished cell proliferation, migration, and invasion in MCF-7 cells [28]. Another study confirmed the tumor-promoting function of PTP1b by identifying that homozygous knockdown of PTP1b (PTP1b−/−) was capable of significantly decreasing or delaying tumor formation, while heterozygous knockdown of PTP1b did not have a significant effect [39]. Hence, PTP1b has been proven to enhance the formation, spread, and aggressiveness of breast cancer (Table 1).

Protein tyrosine phosphatase non-receptor type 2 (PTPN2), also known as T-cell PTP, is another tyrosine-specific PTP that may modulate ER+ breast cancer sensitivity to tamoxifen treatment by lowering the JAK-STAT cancerous signaling pathway [40]. Scientists believe that crosstalk between ER and growth factor signaling pathways plays a role in breast cancer’s resistance to treatment [40,41,42]. Since PTPN2 has been shown to inhibit growth through the PI3K/AKT pathway, it may affect this crosstalk [43,44]. In one study, decreased expression of PTPN2 in ER+ breast cancer was associated with increased expression of nuclear p-AKT, resulting in an overall poorer response to tamoxifen treatment in breast cancer [40]. One study provided a possible mechanism for PTPN2 in breast cancer expressing epidermal growth factor receptors (EGFRs) and ER. ErbB1 (EGFR/HER1) along with downstream Janus kinases and STAT3 are substrates for PTPN2 [43,45,46,47,48,49,50]. Therefore, PTPN2 may directly dephosphorylate ERbB1, leading to inhibition of downstream phospho-JAK and phospho-STAT3 [3,45]. PTPN2 may possibly dephosphorylate JAK and STAT3 directly as well (Figure 1), which could reduce phosphorylation of AKT as a result of JAK-STAT-AKT crosstalk [45,51]. Thus, PTPN2 may serve as a marker that predicts poor patient survival overall and poor response to antihormone treatment when it exhibits loss of function in breast cancer (Table 1).

PTP delta (PTPRD) is a tumor-suppressive, tyrosine-specific, PTP in gastric cancer. Patient data from one study showed that reduced PTPRD expression was associated with diminished survival [52]. Additionally, knockdown of PTPRD induced phosphorylation of STAT3, which heightened survival, proliferation, migration, and invasion as confirmed by phenotypic assays (Figure 1) [52].

SHP-1, PTP1b, PTPN2, and PTPRD are all phosphatases that influence the JAK-STAT pathway in gastric and breast cancer. The mechanisms of these phosphatases must be further studied in other cancers to confirm whether their role is identical in other cell types. For example, SHP-1 and PTP1b impact other pathways and will be discussed in later sections.

## 3. PTPs That Impact SFKs and PTEN

**Figure 2:**
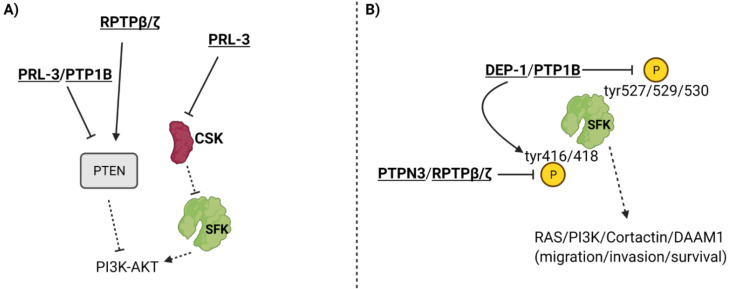
**PTPs that impact SFKs and PTEN.** PTPs can influence PTEN, CSK (Src Kinase inhibitor) (Panel **A**), and phosphorylation of the Tyr527/529/530 inhibitory residues and the Tyr416/418 catalytic residues of the Src family of kinases (Panel **B**). To see how PTEN and SFK function in signaling cascades, refer to Figure 3. For a more in-depth discussion of the mechanism of action for each PTP, refer to Table 2.

PTP1b, which influences JAK-STAT signaling to induce oncogenesis in breast cancer, can also promote breast cancer by dephosphorylating the inhibitory Tyr527 phosphorylation site and activating Src and downstream signaling pathways, which enhances ERbB2 receptor signaling (Figure 2B) [17,18,53]. In this study, ErbB2 was activated with simultaneous knockdown of PTP1b to see how properties such as apoptosis and cell proliferation were affected [53]. They observed that despite ErbB2 activation, the knockdown of PTP1b resulted in increased apoptosis and decreased cell proliferation [53]. In addition to tumorigenesis and proliferation, PTP1b is also crucial for lymph node metastasis, migration, and invasion, as shown by a study that knocked down PTP1b, resulting in reduced migration and invasion [54]. PTP1b modulated these phenotypes by downregulating PTEN expression, thereby upregulating the AKT pathway and increasing the expression of invasion-promoting proteases MMP-2 and MMP-7 (Figure 2A) [54].

PTP1b also exhibits pro-cancerous activity in prostate cancer. In prostate cancer cells, neuroendocrine (NE) cells make up a portion of the tumor and can secrete neuropeptides, growth factors, and other hormones [55,56]. Various studies have proven that hormonal therapy leads to NE differentiation of prostate cancer cells, which can lead to higher metastatic potential [55,57,58,59,60,61]. One study revealed that elevated expression levels of PTP1B can lead to NE differentiation in LNCaP cells [55]. In NE-differentiated LNCaP cells, PTP1B is highly expressed and is exclusively localized to the NE-differentiated cells of the prostate cancer [55]. Further research on PTP1b shows that PTP1b knockdown abrogates migration, invasion, and growth in vitro and in vivo, which highlights its importance as a promoter of prostate cancer [62]. Overall, although the exact mechanism of PTP1B is unknown in prostate cancer, it can be concluded that PTP1B plays an oncogenic role in prostate cancer. Its mechanism may be similar to how it functions in breast cancer, but further research is necessary to uncover how PTP1b elicits pro-cancerous activity in prostate cancer (Table 2). PTP1b is a versatile phosphatase that influences many pathways in breast cancer and possibly prostate cancer to promote tumorigenesis; hence, it must be explored more as a cancer-associated phosphatase that can be used for targeted therapy.

Phosphatase of regenerating liver 3 (PRL-3), also known as PTP4A3, is a dual-specific, non-receptor PTP that exhibits tumorigenic activity in gastric cancer via PTEN-dependent interactions that have been identified in many studies. PRL-3 overexpressing gastric cancer cells exhibited increased p-AKT and downstream matrix metalloproteinase (MMP) expression, which was further confirmed by increased migration and invasion in comparison to control cells [63]. This signaling pathway, along with migration and invasion, was inhibited in PRL-3 overexpressing gastric cancer cells treated with PI3K inhibitor, confirming that PRL-3 acts upstream of the PI3K-AKT pathway to induce its activation [63]. Previous studies strengthened this finding by discovering that PRL-3 inhibits phosphatase and tensin homologue (PTEN) expression, a phosphatase that converts the AKT activating phosphatidylinositol (3,4,5)-triphosphate (PIP3) to phosphatidylinositol (4,5)-bisphosphate (PIP2) [64,65]. Therefore, it is possible that PRL-3 acts upstream of PI3K-AKT by inhibiting PTEN, thus promoting PI3K’s ability to generate AKT activating PIP3 (Figure 2A). It is evident that PRL-3 heightens cancer dynamics (Table 2), validating the need for further research into the inhibition of PRL-3 as a specific therapy in gastric cancer.

PRL-3 is a crucial PTP in breast cancer as well. The PTP4A3 gene is overexpressed in 29% of all basal-like breast cancers and may be a prognostic indicator for poor breast cancer patient survival [66]. A study revealed that PRL-3 elicited the growth, survival, and metastatic progression of triple negative breast cancer in vivo and in vitro [66]. Furthermore, PRL-3 knockdown resulted in G1 cell cycle arrest and apoptosis, which identifies PTP4A3 as being necessary for progression of TNBC [66]. PRL-3 also caused cell cycle arrest in estrogen receptor (ER)-positive breast cancer cell lines, indicating the need for more experiments to confirm PRL-3 as a modulator of cellular dynamics in ER+ breast cancer [66]. The mechanism of PRL-3 has not been thoroughly investigated in breast cancer. However, previous studies probing into the mechanism of PRL-3 in other cancers demonstrated that the phosphatase downregulated the expression of another phosphatase known as phosphatase and tensin homologue (PTEN), leading to the upregulation of the PI3K-AKT pathway through signaling modalities that were previously described (Figure 2A) [64,65]. PRL-3 may also act upstream of Src kinase by downregulating the expression of Src kinase inhibitor c-Srk kinase (Csk), which would lead to the upregulation of many oncogenic pathways downstream of Src (Figure 2A) [20]. Overall, strong evidence suggests that PRL-3 has a tumor-promoting role in breast cancer and induces epithelial-to-mesenchymal transition (EMT), growth, and survival (Table 2).

Receptor protein tyrosine phosphatase beta/zeta (RPTPβ/ζ) is a receptor-type, tyrosine-specific PTP that exhibits tumor suppressor activity in prostate cancer through its interaction with Src and PTEN. A study identified RPTPβ/ζ as an inhibitor of tumorigenesis via its ability to reduce phosphorylation of Src catalytic domain Tyr416 residue and inactivate Src mediated pathways (Figure 2) [67]. Additionally, RPTPβ/ζ reduced phosphorylation of and activated PTEN, which is known to reduce PI3K-AKT signaling in addition to downstream MAPK/ERK signaling (Figure 2) [67]. Receptor protein tyrosine phosphatases (RPTPβ/ζ) and syndecan-3 are pleiotrophin-binding transmembrane receptors [68,69,70]. Experiments revealed that pleiotrophin (PTN), despite being known as a growth factor, inhibits EMT of prostate cancer cells by eliciting PTEN activation and Src inactivation through RPTPβ/ζ [67]. However, PTN promotes EMT and migration by phosphorylating and activating Src and downstream oncogenic signals via syndecan-3 receptor in RPTPβ/ζ knockdown cell lines [67]. The study revealed that RPTPβ/ζ may function as a tumor suppressor by inhibiting tumorigenic signaling pathways and attenuating syndecan-3 signaling (Table 2) [67]. This study focused mainly on changes in Src, PTEN, ERK, and migration. Further studies are required to understand how RPTPβ/ζ affects more key players in the PI3K-AKT and MAPK/ERK signaling pathways in addition to whether RPTPβ/ζ influences apoptosis and cell cycle progression through these pathways [71].

DEP-1/PTPRJ is a tyrosine-specific receptor tyrosine phosphatase that is primarily known for its role as a tumor suppressor [72,73]. Yet, one study found that DEP-1 is expressed more in highly aggressive breast cancer types and may contribute to invasion and migration [74]. Researchers found that when DEP-1 was expressed at intermediate levels, it effectively dephosphorylated Src at Tyr529 inhibitory residue and activated the Src kinase pathway, causing the downstream phosphorylation of Cortactin to promote motile, invasive, and metastatic phenotypes [74]. DEP-1 knockdown resulted in the inhibition of the Src/Cortactin pathway, as represented by reduced phosphorylation of these proteins (Src at Tyr418 catalytic residue) (Figure 2B) [74]. To corroborate these molecular results, the study also revealed that DEP-1 knockdown reduced migration, invasion, and secreted MMP-9 [74]. Though DEP-1 seems to promote cancer progression, scientists discovered through transfection experiments at different DNA concentrations that DEP-1 was most effective at moderate levels, whereas extremely increased or decreased expression of DEP-1 resulted in less Src/Cortactin activation [74]. The same study also highlighted that moderate levels of DEP-1 in breast cancer patients were associated with the highest rates of mortality and cancer relapse, compared to lower and higher expression of DEP-1 in patients [74]. Overall, this study showed that DEP-1, though known for its tumor suppressor role, functions via the Src/Cortactin pathway to enhance metastatic properties in aggressive breast cancer cell types and is linked to poorer prognosis in patients (Table 2). DEP-1 may be a PTP that exhibits varying activity across cell types, though further studies are required to confirm this.

PTPN3, also known as PTPH1, is a non-receptor, tyrosine-specific tumor suppressor PTP in gastric cancer. The mechanism of PTPN3 has been identified in previous research. One study identified that knocking down PTPN3 enhanced production of VEGFA in gastric cancer cells [75]. Incubating human umbilical vein endothelial cells (HUVECs) in conditioned media from PTPN3 KD gastric cancer cells resulted in elevated cell viability, migration, invasion, and more robust tube formation of HUVECs [75]. Another potential mechanism underlying PTPN3 activity may be explained by a study performed on lung cancer cells. PTPN3 was found to dephosphorylate Src at catalytic domain Tyr416 residue and inactivate it, leading to reduced phosphorylation and increased inactivation of disheveled associated activator of morphogenesis 1 (DAAM1). DAAM1 is a key protein in regulating actin dynamics, and inactivation of this protein led to reduced cell migration, invasion, and focal adhesion assembly (Figure 2B) [76,77]. In gastric cancer cells, PTPN3 loss has proven to heighten angiogenesis and tumor metastasis (Table 2). Overall, PTP1b, PRL-3, RPTPβ/ζ, DEP-1, and PTPN3 all modulate SFKs or PTEN in different cancers. PTP1b promotes breast cancer through modulation of the JAK-STAT pathway, Src, and PTEN, while PRL-3 expression augments both breast and gastric cancer.

## 4. PTPs That Affect RTK-Associated PI3K-AKT and Ras-Raf-Mek-Erk Signaling

**Figure 3:**
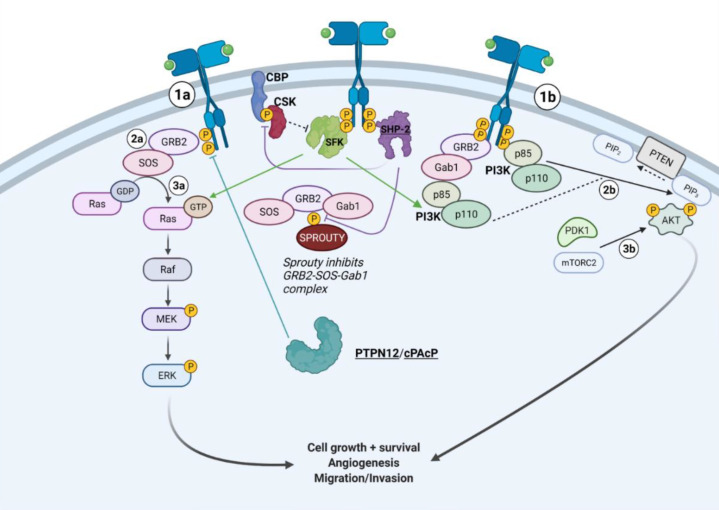
**PTPs that affect RTK-associated PI3K-AKT and Ras-Raf-Mek-Erk Signaling.** The PI3K-AKT pathway and MAPK signaling cascade are downstream of RTKs. The GRB2-SOS-Gab1 complex can enhance PI3K and MAPK signaling. Furthermore, PI3K is recruited to intracellular phosphotyrosine residues of activated RTKs. PTPs dephosphorylate inhibitors (CBP = Csk binding protein) of these oncogenic pathways (SHP-2), or dephosphorylate the RTK itself (PTPN12, cPAcP). For a more in-depth discussion of the mechanism of action for each PTP, refer to Table 3. Adapted from “PI3K/Akt, RAS/MAPK, JAK/STAT Signaling”, by BioRender.com (accessed on 28 September 2021). Retrieved from https://app.biorender.com/biorender-templates (accessed on 28 September 2021).

SHP-2 is a non-receptor, tyrosine-specific PTP that is structurally similar to SHP-1, but is an oncogene that promotes downstream RTK signaling, including RAS pathways in gastroesophageal cancer. Kirsten rat sarcoma virus (KRAS) protein is a Ras GTPase that activates MAPK/ERK signaling (RAS-RAF-MEK-ERK). KRAS WT amplification mutations occur in 17% of esophageal adenocarcinomas and 13% of chromosomal instability gastric cancers [78]. KRAS mutations are common in cancer and can confer treatment resistance. Research shows that MEK inhibition in KRAS-amplified gastric cell lines resulted in a compensatory increase in PI3K-AKT activity and increased active RAS-GTP composition [78]. SHP-2, besides dephosphorylating and inactivating Ras inhibitors, can also promote the binding of growth factor receptor bound protein 2—Son of Sevenless—Grb2-associated binding protein 1 (Grb2-SOS-Gab1) complex to the intracellular portion of receptor tyrosine kinases [11,78,79,80]. The SOS protein, a guanine nucleotide exchange factor, catalyzes the conversion of inactive RAS to active GTP-bound RAS, while Gab1 promotes PI3K signaling (Figure 3) [11,78,79,80,81]. Because SHP-2 is capable of increasing active RAS-GTP, scientists were able to discover that downstream ERK inhibition coupled with SHP-2 inhibition prevented the compensatory increase in RAS-GTP composition and acted synergistically with ERK inhibition to lower cell viability [78]. Another research study further strengthened these findings by highlighting that SHP-2 exhibits stronger expression in poorly differentiated gastric carcinomas [82]. 

SHP-2 also exhibits tumor-promoting activity in basal-like and triple negative breast cancer [83]. Studies in breast cancer confirmed that SHP-2 heightens cancer survival and metastasis [83]. Knocking down SHP-2 inhibited tumorigenesis and metastasis of TNBC cell lines transplanted into mice [83]. Previously mentioned studies on gastric cancer cells have identified potential mechanisms underlying SHP-2-induced oncogenic signaling through enhancement of RTK pathways, which is most likely how SHP-2 functions in breast cancer [11,78,79,80]. In breast cancer cells, SHP-2 was found to enhance RTK activity such as EGFR and FGFR1 as well as downstream MAPK/ERK and PI3K-AKT signaling (Figure 3) [83]. Thus far, SHP-2 has been identified as an oncogene in gastric cancer and breast cancer (Table 3), which warrants further investigation into the RTK-SHP-2 axis and inhibition of SHP-2 as a potential future therapeutic.

SHP-2 upregulates tumor metastasis and cell proliferation in prostate cancer as well [84]. Analysis of an online database showed that elevated PTPN11 (SHP-2) mRNA expression led to poorer patient survival outcomes [84]. Knocking down SHP-2 in cell culture and in vivo models reduced proliferation, tumor formation, migration, and metastasis, whereas knocking down in wild-type SHP-2 elevated these cancerous properties [84]. Further investigation into the mechanism of action highlighted SHP-2′s interaction with partitioning defective (PAR) proteins [84]. Partitioning defective (PAR) proteins complex with atypical protein kinase C (aPKC) to establish cellular polarity and cell-to-cell adhesion [85]. By dephosphorylating PAR3, SHP-2 disrupts PAR3/PAR6/aPKC complex formation and promotes a mesenchymal phenotype of cancer cells [84]. While inhibition of the PAR3/PAR6/aPKC complex may increase proliferation and survival of prostate cancer, PI3K-AKT and Ras activity can also be heightened by SHP-2 as previously described, which may also heavily influence prostate cancer dynamics (Table 3) [11,78,79,80,81]. Therefore, because SHP-2 acts via many mechanisms to promote the PI3K-AKT and MAPK/ERK signaling pathways (Table 3), the SHP-2 oncogene may be a promising target for combination therapies that inhibit RAS and PI3K-AKT.

Another PTP, known as protein tyrosine phosphatase non-receptor type 12 (PTPN12), is a tyrosine-specific, non-receptor type PTP that exhibits tumor suppressor activity in breast cancer by inhibiting certain RTKs including HER2 and EGFR [86]. Previous studies showed that when PTPN12 was silenced in vitro, human mammary epithelial cells (HMECs) increased in colony growth and count, which was further corroborated by increased expression of MAPK/ERK signaling downstream of receptor tyrosine kinases [86]. However, cell transformation was suppressed when PTPN12 was silenced in addition to HER2, EGFR, and MEK inhibition [86]. These results suggested that PTPN12 inhibits transformation in human mammary epithelial cells by directly dephosphorylating and antagonizing HER2, EGFR, and downstream MAPK/ERK signaling (Figure 3) [86]. Further building upon in vitro data, in vivo experiments conducted in NOD/SCID mice indicated that restoring PTPN12 expression in orthotopically transplanted TNBC, via a dox-inducible model, decreased tumor progression and metastasis to the lungs [86]. PTPN12 has been demonstrated to be a potent tumor suppressor in TNBC (Table 3) and could be a key molecular marker used to predict cancer aggression and patient survival.

Human prostatic acid phosphatase (PAcP) is in a unique class of PTPs known as histidine-dependent acid phosphatases, yet also impacts the MAPK/ERK pathway [87]. It is expressed in two forms: its secretory form (sPAcP) and its intracellular form (cPAcP) [88]. While the sPAcP level measured in serum is directly correlated with the development of prostate cancer, cPAcP is minimally expressed in prostate cancer cells [87,88,89,90,91,92,93,94,95]. Specifically, cPAcP levels are inversely associated with cell proliferation, indicating that decreased expression of cPAcP may lead to more aggressive cancers [87,88,89,90,91,92,93,94,95,96]. A study confirmed that highly aggressive prostate cancer cell lines, such as PC3 and DU145, expressed lower levels of cPAcP than moderately aggressive LNCaP and MDA PCa2b cells [87,96,97]. Furthermore, when LNCaP and MDA PCa2b cells were cultured for longer periods of time, as indicated by higher passage numbers, the cells had lower cPAcP expression and elevated proliferation [87,98,99,100]. Research suggests that cPAcP works by dephosphorylating HER2, which leads to the suppression of its tumorigenic activity through the MAPK/ERK pathway (Figure 3) [88]. Hence, cPAcP, a unique PTP in the prostate, exhibits tumor suppressor activity in prostate cancer (Table 3). Overall, SHP-2 seems to be a relatively well-studied phosphatase with a similar function across many cell types, whereas PTPN12 and cPAcP are emerging PTPs that require further studies to elucidate their role in other cancers.

## 5. The Unique Cases of SHP-1 and PTPN12

**Figure 4:**
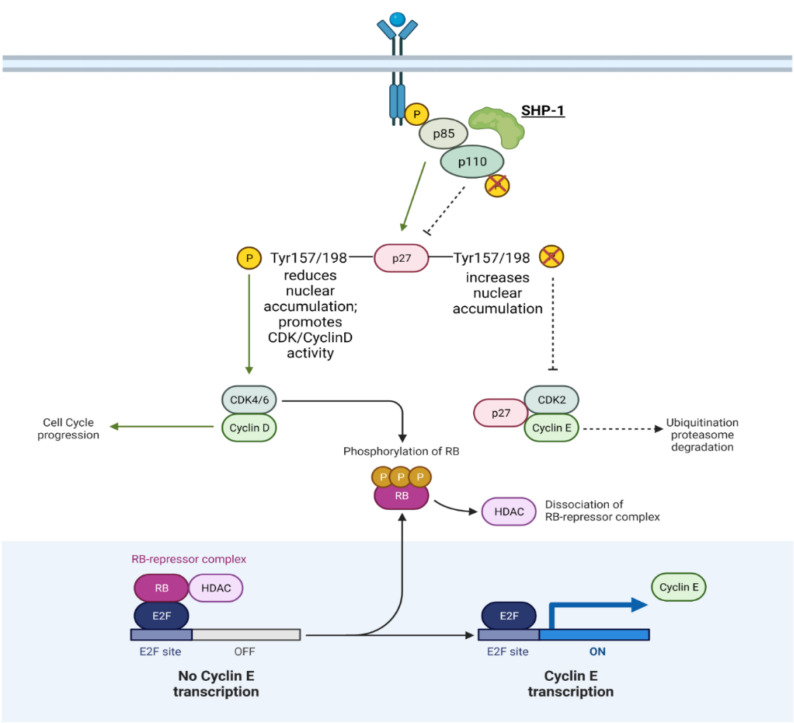
**Mechanism of SHP-1 in prostate cancer.** For a more in-depth discussion of the mechanism of action, refer to Table 3. Adapted from “G1/S Checkpoint”, by BioRender.com (accessed on 28 September 2021). Retrieved from https://app.biorender.com/biorender-templates (accessed on 28 September 2021).

SHP-1 has conflicting tumor-promoting and inhibiting mechanisms in prostate cancer [101,102]. Experiments on SHP-1 in LNCaP prostate cancer confirmed that SHP-1 acted as a tumor suppressor in prostate cancer just as it does in gastric cancer and may have a similar effect on downstream signaling just as it does in gastric cancer (Figure 1) [101]. Conversely, SHP-1 seems to have a different effect based on another study done on PC-3 cells. This study identified that SHP-1 normally interacts with the p85 subunit of PI3K to suppress the inhibitory tyrosine phosphorylation of the PI3K p110 catalytic subunit (Figure 4) [102]. Knocking down SHP-1 increased p110 tyrosine phosphorylation, which inhibits PI3K-AKT signaling [102]. This in turn suppressed phosphorylation of threonine residues 157 and 187 on cyclin-dependent kinase inhibitor p27, leading to reduced degradation and an increase in nuclear accumulation of p27 that suppresses cyclin-CDK complexes from progressing in the cell cycle [102,103,104,105]. Additionally, SHP-1 may directly interact with cyclin-dependent kinase 2 (CDK2) in order to properly translocate the protein to the nucleus for cell cycle progression, a process that is disrupted upon SHP-1 knockdown [102]. Furthermore, SHP-1 knockdown reduced cyclin E expression by inhibiting the expression of CDK6 and subsequent retinoblastoma (Rb) phosphorylation, which means that Rb will bind to and inhibit the E2F family of transcription factors from increasing the expression of key cell cycle promoters such as cyclin E (Figure 4) [102]. Initial data on LNCaP from this study suggested that a similar pathway may operate in LNCaP as well [102]. Therefore, conflicting studies in prostate cancer and gastric cancer highlighted different mechanisms by which SHP-1 may act as an oncogene or tumor suppressor (Table 1 and Table 3). SHP-1 may act as a tumor suppressor in gastric cancer and an oncogene in prostate cancer. However, the conflicting results in prostate cancer studies must be clarified.

SHP-1 is not the only PTP that has different roles in different cancers. PTPN12, although aforementioned as a tumor suppressor in breast cancer, was shown to be highly expressed in prostate cancer (Figure 3) [106]. One study found that high PTPN12 staining was associated with cancerous properties that typically lead to poorer prognosis in patients, such as increased lymph node metastasis, tumor cell proliferation, and prostate-specific antigen recurrence [106]. Researchers revealed that PTPN12 expression was able to predict poor prognosis [106]. Although a mechanism to explain these findings has not been identified, it is evident that PTPN12 may serve as a useful biomarker as an oncogene in prostate cancer, which contradicts the way PTPN12 functions in breast cancer (Table 3) [106]. All in all, research on SHP-1 and PTPN12 suggests that some PTPs can have different functions based on cell type. However, it is unclear whether this is due to the unique pathways that different cells take advantage of or whether it is due to mutations that drive alternative mechanisms of the same PTP. SHP-1 and PTPN12 should be the basis for further investigation on this exciting yet largely unknown phenomenon.

## 6. PTPs That Influence Related Pathways

A set of PTPs influence pathways related to but different than the traditional PI3K-AKT, MAPK/ERK, or JAK-STAT pathways. Receptor-like protein tyrosine phosphatase k (PTPRK) has been identified as a tumor suppressor in central nervous system lymphomas and colorectal cancer [107]. However, one study showed that there is higher PTPRK expression in PC3 cells compared to normal prostate cells [107]. Upon PTPRK knockdown, apoptosis was increased, further confirmed by increased phosphorylated c-Jun N-terminal kinase (JNK), caspase-3, and caspase-8 [107]. Phosphorylation of JNK is known to activate apoptosis, as seen by elevated levels of caspases [107]. Therefore, these data suggest that PTPRK is an oncogene in prostate cancer since it inhibits the JNK pathway and subsequent apoptosis in prostate cancer cells (Table 4) [107].

Low-molecular-weight protein tyrosine phosphatases (LMWPTP) are a group of small-sized PTPs that may exhibit pro-cancerous or anti-cancerous functions [35]. In prostate cancer, LMWPTP is a tyrosine-specific PTP that is overexpressed. One study revealed that high LMWPTP expression was a significant predictor of lower overall survival [108]. Therefore, this study identified LMWPTP as a potential biomarker for predicting prognosis in patients with metastatic hormone-naive prostate cancer [108]. Another study found that increased LMWPTP expression was correlated with cancerous phenotypes such as increased cell migration, decreased cell adherence, and increased anoikis resistance [109]. A proposed mechanism through which LMWPTP promotes cancer is through dephosphorylation of an RTK called EphA2 at its tyrosine 772 residue (Table 4) [110,111]. Prior studies showed that in breast cancer, inactivating EphA2 upregulates the FAK/AKT/Erk signaling, leading to oncogenesis [110,111]. However, because of EphA2′s dual functions as both a tumor suppressor and oncogene in various cancers, further research is required to investigate if a similar mechanism is present in prostate cancer cells [110].

Some PTPs have functions that can promote tumor formation but may aid in sensitizing cancer cells to certain therapies. Protein tyrosine phosphatase H1 (PTPH1), also known as the previously mentioned PTPN3 tumor suppressor in gastric cancer, is a tyrosine-specific, non-receptor PTP that is overexpressed in approximately 50% of breast cancers and promotes growth and survival [112]. However, PTPH1′s oncogenic role in breast cancer does not involve its phosphatase activity [112]. One study showed that PTPH1 binds to the vitamin D receptor (VDR) in the cytosol of breast cancer cells, which causes accumulation of VDR in the cytosol that leads to reduced nuclear localization and transcription [112]. Since VDR is typically growth-inhibitory, a reduction in transcriptional activity results in heightened ER+ and TNBC survival [112]. Despite its oncogenic activity, PTPH1 may aid in the efficacy of antihormone treatment of estrogen receptor positive (ER+) breast cancers [113]. In a different study, scientists found that PTPH1 expressed in MCF-7 cells dephosphorylated ER at its tyrosine 537, which increased ER nuclear accumulation as well as ER degradation [113]. The resulting ER accumulation and degradation in these cells increased their susceptibility to antihormone treatment [113]. Researchers were able to confirm this in vitro by overexpressing PTPH1 in two ER+ cell lines (MCF-7 and T47D) and identifying growth inhibition with two antiestrogen treatments, tamoxifen and fulvestrant, in comparison to control [113]. Therefore, even though PTPH1 exerts oncogenic activity via modulation of VDR dynamics, the phosphatase may be useful as a marker for heightened breast cancer sensitivity to antihormone therapies (Table 4).

## 7. Concluding Remarks

Over the years, much research has been performed on protein tyrosine phosphatases (PTPs). PTPs fall within many categories based on the residue they dephosphorylate, the oncogenic or tumor-suppressive role they have, and protein size and location within the cell. The same PTP may exhibit oncogenic roles in some cancers while having tumor suppressor roles in other cancers. Current evidence suggests that some PTP mechanisms in cancer can be predicted by how they function in a few cancers, while other PTPs may exhibit cancer promoting or suppressing activity based on the unique PTP and cell-type combination. Furthermore, some research has elucidated very detailed mechanisms of how PTPs elicit cancer progression or inhibition, such as in SHP-2 and PTP1b. Other PTPs, such as SHP-1 and PTPN12, have acquired some information as to how they modulate cancer progression but require further research owing to their differing mechanisms in different cancers. Other PTPs have detailed but conflicting reports as to whether they function as promoters or inhibitors of oncogenesis in the same cancer type, such as SHP-1. Therefore, the focus of this review was to highlight the mechanisms of PTPs that were explored in gastric cancer, breast cancer, and prostate cancer, since PTP research is an emerging field with many recent findings. We hope that the explored molecular mechanisms and modulation of cancerous phenotypes underlying PTP activity will lead to further research on how PTPs function in other cancers. Increased treatment efficacy could be achieved by combining PTP-related therapeutics with the wide variety of emerging cancer-associated receptors that are susceptible to immunotherapy [114]. Currently, PTP inhibitors have been explored at the pre-clinical level as well as clinical trials [115,116,117,118,119,120]. However, there are many more PTPs, both tumor suppressor and oncogenic types, that are worth exploring. PTP research in cancer offers remarkable potential to elevate cancer therapeutics to new heights.

## Figures and Tables

**Table 1:** PTPs that regulate the JAK-STAT pathway.

PTP	Classification	Cellular/Molecular Function	Oncogene (O)/Tumor Suppressor (TS)	Figure Illustration
Src homology region 2 domain containing phosphatase 1 (SHP-1)/Tyrosine protein phosphatase non-receptor type 6 (PTPN6)	Non-receptor, tyrosine-specific	Downregulation of JAK-STAT, XIAP, Cyclin D1, MMP-9, VEGF1	TS in gastric cancer	Figure 1
Tyrosine protein phosphatase non-receptor type 1 (PTPN1)/Protein Tyrosine Phosphatase 1B (PTP1B)	Non-receptor, tyrosine-specific	Dephosphorylates STAT3, increases CCL5;	O in breast cancer	Figure 1
Tyrosine protein phosphatase non-receptor type 2 (PTPN2)	Non-receptor, tyrosine-specific	Dephosphorylates ErbB1 (HER1), p-JAK, p-STAT	TS in breast cancer	Figure 1
Protein Tyrosine Phosphatase Receptor Type D (PTPRD)	Receptor-type, tyrosine-specific	Dephosphorylates STAT3	TS in gastric cancer	Figure 1

**Table 2:** PTPs that impact SFKs and PTEN to influence PI3K-AKT, Cortactin, and DAAM1 dynamics.

PTP	Classification	Cellular/Molecular Function	Oncogene (O)/Tumor Suppressor (TS)	Figure Illustration
Tyrosine protein phosphatase non-receptor type 1 (PTPN1)/Protein Tyrosine Phosphatase 1B (PTP1B)	Non-receptor, tyrosine-specific	Dephosphorylates Tyr527 residue of Src (activation); Inhibits PTEN expression	O in breast cancer	Figure 2
PTPN1/PTP1B	Non-receptor, tyrosine-specific	Exact mechanism is unknown. May dephosphorylate STAT3, increases CCL5; Dephosphorylates Tyr527 residue of Src (activation); Inhibits PTEN expression	O in prostate cancer	Figure 1 and Figure 2
Phosphatase of Regenerating Liver 3 (PRL-3)/Protein Tyrosine Phosphatase 4A3 (PTP4A3)	Non-receptor, tyrosine-specific	Inhibits PTEN expression, which heightens PI3K-AKT signaling	O in gastric cancer	Figure 2
PRL-3/PTP4A3	Non-receptor, tyrosine-specific	Not well understood. May heighten Src activity; Inhibits PTEN expression, which heightens PI3K-AKT signaling	O in breast cancer	Figure 2
Receptor protein tyrosine phosphatase beta/zeta (RPTPβ/ζ)	Receptor-type, tyrosine-specific	Reduces Tyr416 phosphorylation of Src and inactivates it; Reduces phosphorylation of and activates PTEN	TS in prostate cancer	Figure 2
Receptor-type tyrosine protein phosphatase eta (PTPRJ)/Density Enhanced Phosphatase 1 (DEP-1)	Receptor-type, tyrosine-specific	Dephosphorylates Src at Tyr529, which increases Src Tyr418 and subsequent Cortactin phosphorylation	O in breast cancer	Figure 2
Protein Tyrosine Phosphatase H1 (PTPH1)/Tyrosine protein phosphatase non-receptor type 3 (PTPN3)	Non-receptor, tyrosine-specific	Dephosphorylates and inhibits Src mediated DAAM1 phosphorylation; Directly inhibits DAAM1 phosphorylation	TS in gastric cancer	Figure 2

**Table 3:** PTPs that affect RTK-associated PI3K-AKT and Ras-Raf-Mek-Erk Signaling.

PTP	Classification	Cellular/Molecular Function	Oncogene (O)/Tumor Suppressor (TS)	Figure Illustration
Src homology region 2 domain containing phosphatase 2 (SHP-2)/Tyrosine protein phosphatase non-receptor type 11 (PTPN11)	Non-receptor, tyrosine-specific	Recruits Grb2-SOS, which catalyzes conversion of inactive Ras to active GTP-Ras; Recruits Grb2-Gab1, which heightens PI3K-AKT signaling	O in gastric cancer	Figure 3
SHP-2/PTPN11	Non-receptor, tyrosine-specific	Recruits Grb2-SOS, which catalyzes the conversion of inactive Ras to active GTP-Ras; Recruits Grb2-Gab1, which heightens PI3K-AKT signaling	O in breast cancer	Figure 3
SHP-2/PTPN11	Non-receptor, tyrosine-specific	Dephosphorylates PAR3, disrupts PAR3/PAR6/aPKC cell polarity/cell-to-cell adhesion complex	O in prostate cancer	N/A
Tyrosine protein phosphatase non-receptor type 12 (PTPN12)	Non-receptor, tyrosine-specific	Dephosphorylates EGFR and HER2 RTKs, which inhibits downstream MAPK signaling	TS in breast cancer	Figure 3
Cellular Prostatic Acid Phosphatase (cPAcP)	Histidine-dependent acid phosphatase	Dephosphorylates HER2 RTKs, inhibits downstream MAPK signaling	TS in prostate cancer	Figure 3
SHP-1/PTPN6	Non-receptor, tyrosine-specific	May function as a TS like in gastric cancer. Oncogenic activity as well: Reduces Cyclin/CDK degradation; Translocates CDK2 to the nucleus; Increases CDK6 expression and Rb phosphorylation → E2F→ Increases Cyclin E	TS/O in prostate cancer	Figure 4
Tyrosine protein phosphatase non-receptor type 12 (PTPN12)	Non-receptor, tyrosine-specific	Unknown	O in prostate cancer	N/A

**Table 4:** PTPs that influence related pathways.

PTP	Classification	Cellular/Molecular Function	Oncogene (O)/Tumor Suppressor (TS)
Receptor-like protein tyrosine phosphatase K (PTPRK)	Non-receptor, tyrosine-specific	Inhibits JNK phosphorylation and subsequent apoptosis	O in prostate cancer
Low-molecular-weight protein tyrosine phosphatase (LMWPTP)	Non-receptor, low molecular weight	May dephosphorylate EphA2 at Tyr772 and upregulate FAK/AKT/ERK signaling	O in prostate cancer
Protein Tyrosine Phosphatase H1 (PTPH1)/Tyrosine protein phosphatase non-receptor type 3 (PTPN3)	Non-receptor, tyrosine-specific	Binds to VDR and inhibits nuclear localization and transcription, enhances tumor survival; Dephosphorylates ER causing accumulation/degradation	O in breast cancer, but sensitizes cancer to anti-hormone treatment

## Abbreviations

Abbreviation	Meaning
PTP	Protein Tyrosine Phosphatase
PTK	Protein Tyrosine Kinase
RTK	Receptor Tyrosine Kinase
LMWPTP	Low Molecular Weight Protein Tyrosine Phosphatase
TNBC	Triple Negative Breast Cancer
ER	Estrogen Receptor
PR	Progesterone Receptor
MCF-7	Human breast cancer cell line (ER+/PR+)
T47D	Human breast cancer cell line (ER+/PR+)
PC3	Aggressive human prostate cancer cell line
DU145	Aggressive human prostate cancer cell line
LNCaP	Moderately aggressive human prostate cancer cell line
MDA PCa2b	Moderately aggressive human prostate cancer cell line
JAK-STAT	Janus Kinase—Signal Transducer and Activator of Transcription
PI3K-AKT	Phosphoinositide 3 Kinase—Protein Kinase B
MAPK signaling pathway	Mitogen-Activated Protein Kinase (Ras-Raf-Mek-Erk pathway)
